# Dislocation Energetics and Pop-Ins in AlN Thin Films by Berkovich Nanoindentation

**DOI:** 10.3390/ma6094259

**Published:** 2013-09-23

**Authors:** Sheng-Rui Jian, Yu-Chin Tseng, I-Ju Teng, Jenh-Yih Juang

**Affiliations:** 1 Department of Materials Science and Engineering, I-Shou University, Main Campus: No.1, Sec. 1, Syuecheng Rd., Dashu District, Kaohsiung City 84001, Taiwan; E-Mail: a0938188197@gmail.com; 2 Centre for Interdisciplinary Science, National Chiao Tung University, Hsinchu 30010, Taiwan; E-Mails: eru7023@gmail.com (I.-J.T.); jyjuang@g2.nctu.edu.tw (J.-Y.J.); 3 Department of Electrophysics, National Chiao Tung University, Hsinchu 30010, Taiwan

**Keywords:** nanoindentation, pop-ins, AlN thin films, transmission electron microscopy

## Abstract

Nanoindentation-induced multiple pop-ins were observed in the load-displacement curves when the mechanical responses of AlN films grown on *c*-plane sapphire substrates were investigated by using Berkovich indenters. No evidence of phase transformation is revealed by cross-sectional transmission electron microscopy (XTEM) and selected area diffraction (SAD) analyses. Instead XTEM observations suggest that these “instabilities” resulted from the sudden nucleation of dislocations propagating along the slip systems lying on the {0001} basal planes and the $\left\{10\bar{1}1\right\}$ pyramidal planes commonly observed in hexagonal compound semiconductors. Based on this scenario, an energetic estimation of dislocation nucleation is made.

## 1. Introduction

Nanoindentation has been widely employed as an important tool to study the mechanical characteristics, including the measurement of hardness and elastic modulus [1,2,3,4,5,6], creep resistance [7] and fracture behaviors [8,9] of various nano-scale materials and thin films. The initial segment in the nanoindentation load-displacement curve usually manifests the elastic behavior of the material under test, whereas the onset of plastic deformation is generally associated with a displacement discontinuity, *i.e.*, pop-in event, during the loading process [10]. It is the difference between the elastic and plastic deformation that allows one to extract prominent information about the mechanical behaviors of the material under investigation by analyzing various segments of the load-displacement curves obtained. Assuming a spherical indenter tip geometry, the elastic behavior can be analyzed by the elastic contact deformation relationship given by the Hertzian elastic contact model [11], as follows:

(1) $$P=\frac{4E_{r}}{3}\sqrt{Rh}$$

Here P, R and h are representing the applied indentation load, the radius of indenter tip, and the corresponding indentation depth, respectively. The reduced elastic modulus $E_{r}$, is given by:

(2) $$E_{r}={\left(\frac{1-v_{f}^{2}}{E_{f}}+\frac{1-v_{i}^{2}}{E_{i}}\right)}^{-1}$$

where v is the Poisson’s ratio, E is the Young’s modulus, and the subscripts *i* and *f* are denoted as the indenter tip and indented films, respectively. For a diamond indenter tip, $E_{i}$ ≈ 1141 GPa, $v_{i}$ = 0.07 and, for AlN thin film, $v_{f}$ = 0.3 [12], $E_{i}$ ≈ 244 GPa [13] are adopted in this work. A significant deviation of the experimental data from the Hertzian relationship is usually attributed to the onset of plastic deformation occurring when the load applied to the material exceeds certain values [14].

In previous studies, no pop-in [15] or multiple pop-ins behaviors [13] were observed in the load-displacement curves of AlN thin films during nanoindentation. However, since the onset of nanoscale plasticity and mechanical properties are strongly influenced by various factors, such as crystal orientation [16] and temperature [17] during nanoindentation measurements, the interplays between the observed pop-in events and the onset of micro/nanoscale plasticity for AlN thin films remain elusive. Herein, the pop-in behaviors and mechanical characteristics of AlN thin films derived from nanoindentation with a Berkovich indenter are discussed in conjunction with the observations of cross-sectional transmission electron microscopy (XTEM). The use of a Berkovich indenter not only will reflect the more realistic situations that might be encountered in real applications but also will result in much higher local pressure with similar load, and hence, will be helpful in clarifying the issue of indentation-induced structural deformation [18]. Since the first pop-in typically appears at indentation depth below 100 nm, at such a low depth (within the limit of the same size as the diameter of the tip apex) Equations (1) and (2) are still a reasonable approximation also for the Berkovich indenter. The correlation between the observed Berkovich nanoindentation-induced dislocation nucleation and propagation on AlN thin films and the number of dislocation loops formed in the pop-in phenomenon are also estimated based on the classical dislocation theory [19].

## 2. Experimental Details

AlN thin films with thickness of 1 μm were deposited on the *c*-plane sapphire substrates by using a homemade helicon sputtering system. The detailed growth procedures of the present AlN/*c*-sapphire films can be found elsewhere [20].

**Figure 1:**
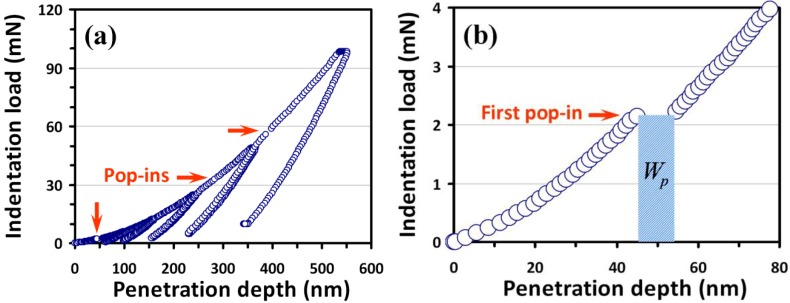
(**a**) Load-displacement curve measured by Berkovich indenter on AlN thin films at the indentation load of 100 mN and (**b**) the corresponding first pop-in event (see the arrow) from an expanded region of (**a**), where the plastic strain work is denoted as, *W_p_* (critical loading times the sudden incremental displacement at constant load).

Nanoindentation tests were performed on a MTS NanoXP^®^ Nanoindenter system (MTS Cooperation, Nano Instruments Innovation Center, Oak Ridge, TN, USA) with a diamond pyramid-shaped Berkovich-type indenter tip. The radius of curvature of the tip is 50 nm. The area shape of the Berkovich indenter was calibrated using fused silica. The frame stiffness and thermal drift were corrected for all measurements. The thermal drift was kept below ±0.05 nm/s for all indentations considered in this work. The same loading/unloading rate of 10 mN/s was used. Moreover, in order to reveal the role played by the nucleation and propagation of dislocations in indentation-induced deformation, cyclic nanoindentation tests are also performed in this work. The cyclic nanoindentation tests were performed in a sequence described as follows. Firstly, the indentation loading was applied to the set maximum load and unloaded by 90%. Then, reload the indenter to the maximum load and unload it by 90% again. At this stage, the indenter was held for 10 s at 10% of the maximum load for thermal drift correction. After that, the indenter was completely unloaded to complete a cyclic nanoindentation test for a certain targeted maximum load. Consequently, a typical load-displacement curve comprises four cyclic tests to maximum loads of 6, 12, 25, 50 and 100 mN and is shown in Figure 1a. At least 30 indents were performed and the nanoindentations were sufficiently spaced to prevent mutual interactions. The maximum penetration depth at maximum load is around half the film thickness. At higher loads, the harder sapphire substrate is expected to influence the mechanical behavior of the film.

After indentation to a maximum load of 100 mN, the XTEM samples of AlN films were prepared by the lift-out technique using a dual-beam focused ion beam station (FIB, FEI Nova 220, Hillsboro, OR, USA) with Ga ions at 30 keV. Prior to milling, the FIB was used to deposit ~1 µm-thick layer of Pt to protect AlN thin films surface. The XTEM lamella was examined in a JEOL 2010F TEM (JEOL Ltd., Tokyo, Japan) operating at 200 kV with a point-to-point resolution of 0.23 nm and a lattice resolution of 0.10 nm.

## 3. Results and Discussion

The X-ray diffraction results (not shown here) and XTEM image (see below) confirmed that the hexagonal wurtzite structured AlN films obtained by the present deposition process are of high crystalline quality similar to those obtained previously [20]. Figure 1a shows the typical load-displacement curve obtained for AlN thin film with a maximum indentation load of 100 mN. It is evident that there exist plastic deformation irregularities along the load-displacement curve, which are characterized by multiple discontinuities occurring at certain penetration depths (indicated arrows), referred to as multiple “pop-ins”. This observation is consistent with our previous study on AlN films grown on a Si(111) substrate [21], but is in contrast to that reported by in the previous study of AlN thin films [15], where no “pop-in” was observed. This discrepancy might be due to the detailed film growth conditions, which can lead to very different film microstructures, as well as different indentation conditions. On the other hand, it is also noted that the multiple pop-ins displayed by the current AlN thin films is similar to phenomena previously seen in other compound semiconductor materials with hexagonal crystal structure [3,5], wherein the multiple slip systems available for indentation-induced dislocation nucleation and propagation have been identified as the primary reason giving rise to the observed multiple pop-in phenomena. The fact that there is no “pop-out” event on the unloading curves (see Figure 1a), further suggests that, unlike that observed in indented AlN/Si(111) films [21] and silicon [22], phase transitions probably are not occurring either in the AlN film or in the underlying sapphire substrate in the present case. In order to delineate the complex deformation processes involving dislocation nucleation and propagations, direct microstructural investigations with the aid of XTEM techniques are indispensible.

**Figure 2:**
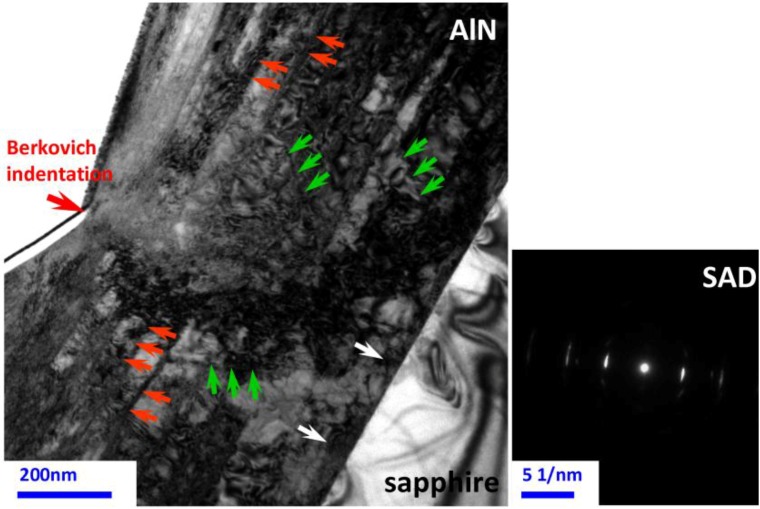
A bright-field XTEM image in the vicinity under the Berkovich indent applied on the AlN thin film with an indentation load of 100 mN. In addition, the selected area diffraction (SAD) pattern results of indented AlN thin film underneath the Berkovich nanoindentation.

Figure 2 is a bright-field XTEM image of the present AlN/*c*-sapphire thin film after being indented at a load of 100 mN. It clearly shows that the film is highly epitaxial with no feature of columnar grain structure except threading dislocations (see the white arrows). It also shows that the deformation features underneath the indented spot are primarily manifested by dislocation activities within the film, namely slip bands aligned parallel to the {0001} basal planes (see the orange arrows). In addition, slip bands oriented at ~60° to the sample surface are also found (see the green arrows). The ~60° slip bands, presumably originating from dislocations gliding along the $\left\{10\bar{1}1\right\}$ pyramidal planes, are, however, distributing in much shallower regions near the contacting surface. This indicates that a much higher stress level is needed to activate these slip systems as compared to the ones along the basal planes. The distorted slip bands and the extremely high dislocation densities at the intersections indicate highly the strained state of the material. Furthermore, the selected area diffraction (SAD) analysis of the deformed area shown in the right panel of Figure 2 indicates that the AlN film is indeed single crystalline-like and no sign of any halo rings is observed, except for the slight distortion and streaking on the diffraction spots due to severe strain. These features further confirm that no phase transformation and amorphization were occurring during the entire indentation process.

Based on the above microstructural observations, it can be assumed that in the present case the indentation-induced plastic deformation prior to the pop-in event is associated with the individual movement of a small number of newly nucleated and/or pre-existing dislocations. As the number of dislocations is increased with increasing loading, they start to entangle with each other, resulting in effective dislocation pinning, which, in turn, hinders the dislocation propagations. Consequently, tremendous shear stress is quickly accumulated underneath the indenter tip. When the local stress reaches some threshold level, a burst of collective dislocation movement on the easy slip systems is activated, leading to a sudden release of local stress and a pop-in event on the load-displacement curve.

Within the framework of this scenario, there are, at least, three possible mechanisms responsible for this behavior. First, the specific hexagonal lattice structure of AlN offers the possibility of interactions and pinning between dislocations gliding along the pyramidal and basal planes. Second, the usual “slip-stick” behavior [23] can also prevail by the interactions between the as-grown dislocations and those being punched out by nanoindentation loading. Finally, the behavior could just be a consequence of punching out of the threading dislocations above the critical threshold energy by Berkovich nanoindentation. That is, the threading dislocations originally existing in the AlN thin films could respectively display sudden propagation when they acquire enough energy from the deformation, resulting in the multiple pop-in behaviors. The fact that the phenomena are observed only in hexagonal structured materials strongly suggests that the first mechanism might be of primary importance.

In the scenario of the dislocation nucleation and propagation mechanism, the first pop-in event reflects the transition from perfectly elastic to plastic deformation. That is, it is the onset of plasticity in AlN thin film. Under this circumstance, the corresponding critical shear stress ($\tau_{\max}$) under the Berkovich indenter at an indentation load, $P_{c}$, where the load-displacement discontinuity occurs, can be determined by using the following relation [11]:

(3) $$\tau_{\max}=0.31{\left(\frac{6P_{c}E_{r}^{2}}{\pi^{3}R^{2}}\right)}^{1/3}$$

where R is the radius of indenter tip. The $\tau_{\max}$ for AlN thin film has been reported to be about 5.4 GPa [13]. We assume that this $\tau_{\max}$ is responsible for the homogeneous dislocation nucleation within the deformation region underneath the indenter tip.

It has been proposed that the first pop-in is associated with the homogeneous dislocation nucleation mechanisms during nanoindentation [14,24,25,26]. According to the classical dislocation theory [19], the shear stress required to initiate plastic deformation depends on the energy required to generate a dislocation loop. The free energy of a circular dislocation loop of radius, *r*, is given by:

(4) $$F=\gamma_{dis}2\pi r-\tau b\pi r^{2}$$

where $\gamma_{dis}$, *b* and τ are denoted as the line energy of the dislocation loop, the magnitude of Burgers vector (~0.311 nm) [12] and the external shear stress acting on the dislocation loop, respectively. The first term on the right-hand side of Equation (4) describes the energy required to create a dislocation loop in a defect-free lattice and is equal to the increase in lattice energy due to the formation of a dislocation loop. The second term gives the work done by the applied stress (τ) as a result of the Burgers vector displacement and reflects the work done on the system to expand the dislocation. The line energy for a circular loop ($\gamma_{dis}$), which results from the lattice strain in the vicinity of the dislocation for $r>r_{core}$, is given by [19]:

(5) $$\gamma_{dis}=\frac{Gb^{2}}{8\pi }\frac{2-v_{f}}{1-v_{f}}\left[\ln\left(\frac{4r}{r_{core}}\right)-2\right]$$

where G is the shear modulus of AlN thin film (=$E_{f}/2(1+v_{f})$ ≈ 97.4 GPa) and $r_{core}$ is the radius of dislocation core. By using Equations (3) and (5), Equation (4) can be rewritten as:

(6) $$F=\frac{Gb^{2}r}{4}\left(\frac{2-v_{f}}{1-v_{f}}\right)\left(\ln\frac{4r}{r_{core}}-2\right)-\pi br^{2}\tau_{c}$$

Equation (6) relates the material properties and experimentally observed pop-in load ($P_{c}$) to the free energy responsible for dislocation nucleation; in addition, $\tau_{c}$ is the resolved shear stress of $\tau_{\max}$ on the active slip system and is usually taken as half $\tau_{\max}$ [27]. The nucleation of a dislocation loop is similar to the nucleation of a spherical new phase within a homogeneous matrix, where the corresponding function *F* contains terms with first and second power of *r*. *F* has a maximum at a critical radius (*r_c_*) above which the system gains energy by increasing *r*. According to Equation (6), this maximum energy is decreased with increasing load and a pop-in, *i.e.*, homogeneous formation of circular dislocation loop, becomes possible without thermal energy at F=0 [28,29]. The F=0 condition allows $\tau_{c}$ to be found Equations (4) and (5) giving:

(7) $$\tau_{c}=\frac{2\gamma_{dis}}{br}$$

Noting that $\tau_{c}$ has a maximum when $d\tau_{c}/dr=0$, Equations (5) and (7) yield:

(8) $$r_{c}=\frac{e^{3}}{4}r_{core}\approx 5\;r_{core}$$

Here, the value of $r_{core}\approx 0.42$ nm is calculated and compared with the previous experimental TEM observations as 8-atom ring structure with diameter in the order of ~0.5 nm in AlN thin film [30]; thus, $r_{c}\approx 2.1$ nm can be calculated to obtain from Equation (8).

The number of dislocation loops formed during the first pop-in can, thus, be estimated from the work-done associated. From the shaded area depicted in Figure 1b, this work-done is estimated to be ~20 × 10^−12^ Nm, implying that ~5 × 10^6^ dislocation loops [obtained from Equation (6)] with critical diameter formed during the pop-in event. This number is low and is consistent with the scenario of homogeneous dislocation nucleation-induced pop-in, instead of activated collective motion of pre-existing dislocations [14]. When the total dissipation energy, namely the area between the loading and unloading curves shown in Figure 1a, is taken as the energy to generate dislocations with critical radius, as high as ~3 × 10^8^ dislocation loops may be formed within a loading-unloading curve. Although it is not realistic to assume that all the dissipated indentation energy was entirely transferred to generate dislocation loops, the estimation has, nevertheless, provided an upper limit for the number of dislocation loops with critical radius in the initial state. Finally, we note that the first pop-in event used in the calculations described above occurs at a load typically around 2 ± 0.5 mN (obtained from more than 20 load-displacement curves), which is about 50 times lower than the maximum load, thus the substrate influence can be neglected. Moreover, the sudden incremental displacement associated with the first pop-in was about 10 ± 3 nm, which should give an approximate variation range of the dislocation generation energetics discussed here.

## 4. Conclusions

In summary, a combination of Berkovich nanoindentation and XTEM techniques was carried out to investigate the Berkovich nanoindentation-induced pop-in effect in AlN thin films grown on sapphire substrates. The main findings are briefly summarized as followings: Multiple pop-ins phenomena are found in the load-displacement curve on loading, while no pop-out is observed during unloading cycle. In addition, the nanoindentation-induced mechanical deformation is due primarily to the generation and propagation of dislocations gliding along the {0001} basal planes and the $\left\{10\bar{1}1\right\}$ pyramidal planes specific to the hexagonal structure of AlN thin film from XTEM observations. The number of the nanoindentation-induced dislocation loops estimated from the work-done within the course of the pop-in event suggested that the pop-in was mainly due to homogeneous nucleation of dislocations. Similar to many hexagonal-structured semiconductors, the primary deformation mechanism for AlN thin film is nucleation and propagation of dislocations. Based on the scenario, the nanoindentation-induced generation of dislocation loops associated with the first pop-in event was estimated to be in the order of ~5 × 10^6^ with a critical radius (*r_c_* ≈ 2.1 nm). The obtained dislocation density is relatively low and is consistent with the scenario of homogeneous dislocation nucleation-induced in the first pop-in event.
